# Development of the ‘Attentive Visitors’ workshop supporting community volunteers in their palliative care signposting role

**DOI:** 10.1016/j.puhip.2025.100663

**Published:** 2025-10-04

**Authors:** Sabet Van Steenbergen, Steven Vanderstichelen, Else Gien Statema, Luc Deliens, Sarah Dury, Kenneth Chambaere

**Affiliations:** a End-of-Life Care Research Group, Vrije Universiteit Brussel (VUB) & Ghent University, Ghent, Belgium; b Department of Public Health and Primary Care, Ghent University, Ghent, Belgium; c Compassionate Communities Centre of Expertise, Vrije Universiteit Brussel (VUB), Brussels, Belgium; d Department of Family Medicine and Chronic Care, Vrije Universiteit Brussel (VUB), Brussels, Belgium; e Department of Educational Sciences, Vrije Universiteit Brussel (VUB), Brussels, Belgium; f Society and Ageing Research Lab, Vrij Universiteit Brussel (VUB), Brussels, Belgium

## Abstract

**Background:**

Community volunteers in palliative care often notice needs that healthcare professionals and family caregivers miss, playing an important signposting role in several settings. However, community volunteers often still go unnoticed, are frequently insufficiently supported and their important contributions underutilized. In order to fulfill a signposting role, community volunteers need knowledge about palliative care needs and community resources; and they should have good relational, communication and observation skills. Training modules specifically aimed at these skills and knowledge can play an important supportive role for community volunteers.

**Objectives:**

To develop a training to support community volunteers in their signposting role for palliative care of community residents.

**Study design:**

A formative intervention development study.

**Methods:**

The training was developed in the form of an interactive workshop. It's materials and protocol were designed and produced based on the following methods: review of available educational resources for community volunteers, 16 program design meetings with a psychologist trainer and 3 stakeholder advisory board meetings.

**Results:**

The learning objectives are to increase awareness and knowledge of the volunteer role and signposting function, palliative care needs and signals, community resources, and increase self-efficacy in communication of care signals with the community resident and with healthcare professionals. The ‘Attentive Visitors’ workshop consists of a didactic session (7,5 h) focusing on developing knowledge, skills and self-efficacy, and a follow-up session (3 h) focusing on reflection and the exchange of volunteers' knowledge and experiences. Case discussions, reflection exercises and role plays are used to enhance volunteers' insights and skills related to recognizing, describing, responding to and communicating patient needs to healthcare professionals.

**Conclusion:**

The evidence-informed Attentive Visitors Workshop aims to support community volunteers in their signposting role and to improve the connection and information exchange between volunteers and healthcare professionals. Future research will pilot and evaluate the workshop's effectiveness, acceptability and feasibility.

## 1 Introduction

Community volunteers hold a unique and pivotal role in bridging the gap between healthcare professionals, community residents with palliative care needs, and informal caregivers [1]. Operating independently of professional services, these volunteers provide complementary care through home visits, addressing a range care and support needs [1,2]. By offering companionship, combating loneliness, and alleviating the burden on informal and professional caregivers, volunteers enhance the wellbeing of individuals with chronic and palliative care needs [3]. Their proximity to residents allows them to observe and identify needs and wishes that otherwise go unnoticed to healthcare professionals, thus playing a vital signposting role (i.e., identifying and helping to communicate patient's care needs and wishes to healthcare professionals) [[4], [5], [6]]. Given the ageing global population, the growing burden on healthcare systems [7], and the projected rise in individuals requiring palliative care [8] and dying at home [9], the role of community volunteers will become increasingly vital.

According to Allen Kellehear's 95 % rule, individuals with life-limiting illnesses spend 95 % of their time outside of formal healthcare settings, depending on informal support from family, friends, colleagues, neighbors, volunteers and online communities [10]. For individuals with limited social networks, community volunteers are crucial in bridging gaps in support. Evidence suggests that volunteer support positively influences quality of life, social connectedness and cohesion, and illness experience for those at the end of life [[11], [12], [13], [14], [15], [16]] and during bereavement [[17], [18], [19]]. Research by Abel et al. describes community volunteer support as an important component of an emergency admission reducing ‘compassionate community’ intervention [20] (such interventions are health-promoting community-based approaches to increase community support for and reduce suffering related to experiences of serious illness, death, dying and loss [21]). Additionally, among families, the support of volunteers is appreciated [[22], [23], [24], [25]], and those receiving more volunteer hours have rated quality of palliative care higher [26].

Despite their unique position, valuable contributions and the time they spend with community residents, it is uncommon for volunteers and healthcare professionals to engage in communication, information exchange and collaboration [5]. Studies show that, while volunteers in specialist palliative care services are often well supported and trained in basic palliative care knowledge and communication with healthcare professionals, this is much less common among community volunteers [27,28]. This notwithstanding, community volunteers are actively involved in supporting people faced with end-of-life challenges and palliative care needs [28]. A previous study indicated that for volunteers to recognize, describe and signpost care needs towards healthcare professionals, they need understanding of various palliative care needs, familiarity with community resources, and well-developed relational, communication, and observation skills [1]. Targeted training and support programs for community volunteers could better enable them to fulfill signposting roles and work more effectively alongside healthcare professionals [1].

Brighton et al. [29] investigated the learning preferences of hospital volunteers engaged in palliative and end-of-life care training. Hospital volunteers valued learning from peers and palliative care specialists using interactive teaching methods including real-case examples and role plays [29]. A chance to refresh training insights at a later date was suggested to enhance the learning experience. Due to the possible emotional challenges associated with volunteering in palliative care, it is recommended that support, the sharing of experiences, and intervision to be integrated into the training of volunteers in this field [29]. In the same line, other studies reported on the desire of volunteers for ongoing education and the opportunity to connect with others who understand the challenges of their role [[13], [14], [30]]. These insights regarding learning preferences are likely transferable to the community volunteers in palliative homecare, given the lack of defined guidelines regarding training or mentoring in this setting. Furthermore, a number of training programs have been developed with the aim of addressing these learning preferences and providing support to community volunteers in a number of key areas, including communication skills [29,[31], [32], [33], [34]], understanding grief and bereavement [33], spiritual diversity [31], common symptoms [32], self-care [29,31,32], and care navigation [13,14]. Notable examples include ‘Compassionate Neighbors’ [35], ‘Compassionate Communities Connectors’ [36,37], ‘NavCare/EU Navigate’ [13,14], ‘EASE’ [38] or ‘iLIVE’ [39]. However, these multi-day training programs strengthen knowledge and skills of community volunteers, they do not focus on signposting.

To address this gap, we developed the Attentive Visitors workshop for community volunteers who visit community residents with chronic and/or palliative care needs at home, to support them in their signposting role. This paper reports on the development process, the content and training materials used in the Attentive Visitors workshop.

## 2 Methods

### 2.1 Design

**Fig. 1:**
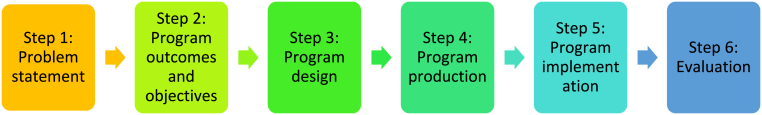
Different steps of the Intervention Mapping Protocol (IMP) [40].

This study follows the Intervention Mapping Protocol (IMP) [40]. This paper is part of a broader research project that aims to develop, test and evaluate a training program that supports community volunteers in their signposting role. We follow the Intervention Mapping Protocol (IMP), an approach to the development of interventions aimed at the successful change of behavior [40]. (See Fig. 1.)

Steps 1 and 2 were reported elsewhere [1]. The current paper focuses on step 3: program design and step 4: program production. See Table 1 for an overview of the methods used for the development the workshop program.

***Step 3: Program Design***: The workshop was designed in collaboration with a professional trainer (ES) and our stakeholder advisory board. Box 1 gives an overview of the various backgrounds of the stakeholders involved.

**Table 1:** Overview of methods used for the development of the workshop program [40].

Step 3: Program design	- Non-exhaustive review of available Flemish educational resources for community volunteers regarding palliative care, singposting and communication − 16 Program design meetings with psychologist trainer and members of the project group − 3 Stakeholder advisory board (N = 9) consultation meetings

Step 4: Program production	- Program validation: face and content validity testing - Production of the workshop materials − 3 Stakeholder advisory board (N = 9) consultation meetings

Theory- and evidence-based behavior change methods were selected based on the learning objectives formulated in steps 1–2 [1,40] and existing Flemish training programmes were reviewed for inclusion. Flemish training programs were identified through input from stakeholder advisory board, a web search, reviewing existing training modules available within the research group and from our stakeholders, and contacting organizations that offer training with overlapping content. The resulting materials were then evaluated using a list of criteria presented in Box 2. Existing materials that fit the criteria were incorporated into the workshop.

Box 1 **Members of Stakeholder Advisory Board of volunteer training experts and coordinators**

- Palliative Care Network coordinator
- Palliative Care Policy officer
- Nurse expert in Pain and Comfort
- Educational officer and volunteer coordinator of a Community-based homecare organisation
- Community-based homecare organisation head of Department
- Volunteer coordinator of a palliative daycare centre
- Coordinator of a family caregiving support organisation
- Palliative Care Specialist and former hospice coordinator
- General Practitioner

Alt-text: Box 1

Box 2 **Selection criteria for workshop methods and materials**

During the development and selection of workshop methods and materials, the following selection criteria were considered to evaluate their appropriateness for integration into the Attentive Visitors workshop:

1. *Can the workshop method contribute to achieving one or more learning objectives?* Specific learning objectives were established prior to the workshop, based on the qualitative research conducted in steps 1 and 2 (Problem Statement & Program Outcomes and Objectives), which determine the focus of the workshop. The overall topic of the workshop is 'detecting (palliative) care needs,' within which specific sub-goals were formulated.
2. Does the chosen method fit the type of outcome it aims for?
3. *Does the chosen method align with the target audience and group size*?
4. *Does the sequence and variation of workshop methods ensure continuous attention and engagement?* Variation must be present in several factors, such as using different senses and intelligences, but also by having participants work alternately in pairs, then in groups of three, and finally as a whole class. This way, different approaches can be accommodated, thereby maintaining the attention of participants.
5. Does the method leave room for acknowledgment and build further on existing knowledge and skills of the participants?

Alt-text: Box 2

Where gaps in the existing training options were identified, new workshop components were developed (See Table 1) using ES's input and expertise, as well by aligning learning objectives with appriatiote methods. The workshop was designed as a modular package, with each module covering one learning objective to construct a comprehensive reference workshop course.

***Step 4: Program Production***: In Step 4, the content and methods developed in Step 3 were put together into a concrete program [40]. We produced a description of the scope and sequence of the components of the intervention, program materials, and program protocols. The workshop was then tested internally and the composition was fine-tuned in consultation with our stakeholders, ensuring that the workshop fit the learning objectives, was relevant for community-based volunteers visiting people at home), and that they met the standards of excellence expected in the field. The required workshop materials and exercises were crafted and honed to create a workshop program. Table 1 presents the methods used in Step 4.

## 3 Results

### 3.1 Program design (Step 3 of the IMP)

#### 3.1.1 Review of available educational resources for community volunteers

We identified 6 of 34 reviewed training materials (modules or working methods) available and used in existing Flemish training programs. See Table 2 provides an overview of their goals and which elements were considered useful for the Attentive Visitors workshop.

**Table 2:** Remaining training programs, training modules, and exercise after the review.

Reviewed training programs, modules or exercises	Goals	Elements (core ideas, exercices or working methods) included in the Attentive Visitors workshop
Basic training in palliative care for volunteers, offered by the Palliative Networks in Flanders [41]	• Acquiring basic knowledge regarding palliative care • Acquiring basic skills in dealing with patients and family • The emphasis is mainly on attitude formation and awareness of one's own attitude towards dying, loss and mourning	The following elements were adapted and included: • Exercise to practice conversation techniques regarding communication with patient and environment • Exercises to become aware of one's own boundaries

Dealing with existential questions [42]	- Awareness of own life questions in relation to meaning, experience of meaning and loss of meaning - Awareness of own listening attitude - Awareness of own boundaries and communication of one's own boundaries	The following elements were adapted and included: exercise on awareness and formulation of one's own boundaries based on a case and guiding questions

Volunteers as referrers, offered by Christian Sickness Fund (CM) [43]	- Awareness of volunteer role - Awareness of own boundaries and communication of these boundaries	The following core ideas were inlcudes in the case studies: • As a volunteer, do not act in the place of the person seeking help, but leave room for his own initiative and commitment. • As a volunteer, you are confronted with situations/questions for which you do not know how to solve them, and from that realization, appeal to relevant stakeholders. • Volunteers are not professional care providers. • Referral does not mean rejection.

SEE ME training [44]	- Seeing the person behind the patient - Seeing care as more than physical or medical care - Recognizing social and meaning-making needs - Teaching people to see the talents and strengths of the oldest citizens	The following elements were adapted and included: • Use of SEE ME in depth questions • Case study exercise regarding awareness of the social and meaning needs of older adults • Explore and recognize basic needs based on talent scan and in-depth questions

An Katz's communication model (4O model) [45]	- Structuring communication in simple steps	The following elements were adapted and included: application of the conversation model to cases (opening statement, open question, reviewing options and following up on concrete actions)

Principles Photovoice [46] and Photo Elicitation [47]	- Use of generated photos to generatie ideas and experiences	The following elements were adapted and included: use of images to stimulate relevant reflection about their volunteer role Icebreaker exercise

Notable gaps in existing training programs were: (1) lack of specific focus on the community context, and (2) lack of explicit focus on communication and signposting of care needs to the community residents or healthcare professionals. New components to the workshop were therefore designed to address these gaps.

#### 3.1.2 Program design meetings with psychologist trainer

A total of 16 meetings (occurring on a weekly basis) were held between SVS and ES from April 2022 to November 2022 to discuss the content, structure and time investment of the workshop. Workshop materials were developed that focused on balancing knowledge, understanding, and skills exercises. Opportunities for reflection and experience sharing were incorporated throughout the workshop.

#### 3.1.3 Stakeholder advisory board consultation meetings and project group meetings

The design of the workshop was discussed with our stakeholder advisory board and our scientific project group (SVS, SV, ES, SD, KC, LD). The stakeholder advisory board met on 3/5/22, October 24, 2022, and August 24, 2023 and helped develop the workshop materials, including a palliative care knowledge quiz and two persona cases. A palliative care specialist ensured the cases' relatability. Pre-defined case studies allow trainers maintain control and reduce participant burden. The stakeholders recommended emphasizing the importance of addressing care and support need signals with the resident first, and of the resident's consent prior to contacting a healthcare professional directly. Additionally, it was agreed that the signposting role should not be framed as mandatory, but the workshop should enable volunteers who want to take up this role. Finally, stakeholders urged us to explicitly call the workshop a *workshop*, as it emphasizes interaction and experience-sharing. Table 3 provides an overview of the different learning objectives in the workshop, their corresponding sub-goals and methods.

**Table 3:** Learning objectives, sub-goals and corresponding methods used in the Attentive Visitors Workshop.

	Learning objective	Sub-goal	Method
**PART 1: didactic workshop (7,5 h)**	Participants will know their fellow participants better.	- Icebreaker - Getting to know eachother	Introduction by photos **(**Appendix I), inspired by Principles of Photovoice [46] and Photo Elicitation [47]
	Participants will have increased knowledge about palliative care.	- Reviewing and enhancing basic knowledge about palliative care - Activating and improving knowledge about palliative care, including it's various domains of care needs	Quiz **(**Appendix II**),** inspired by basic training in palliative care for volunteers, offered by the Palliative Networks in Flanders [41]

	Participants will have increased awareness of the volunteer role and the signposting function.	- Increasing awareness of one's own volunteer role in palliative care and the potential signposting function of volunteers - Gaining insights into the importance of and how to maintain one's own boundaries	Group discussions, inspired by: - Basic training in palliative care for volunteers, offered by the Palliative Networks in Flanders [41] - Course book on dealing with existential questions [42] - Volunteers as referrers, offered by Christian Sickness Fund (CM) [43]

	Participants will have increased knowledge palliative care needs, and increased skills and self-efficacy in identifying available community and professional care resources.	- Increasing understanding and knowledge of the various domains of palliative care and care needs and connecting them to different forms of support - Gaining insight into one's own tendencies and blind spots, and understanding someone's needs from the perspectives of their dreams, wishes and hopes - Increasing knowledge about available support initiatives	- Case study **(**Appendix III**)** and Group discussion, inspired by the SEE ME training [44]

	Participants will have increased awareness of boundaries and engaging other community resources.	- Gaining insights into the importance of and how to maintain one's own boundaries - Increasing knowledge about identifying support initiatives in the neighborhood	Group discussions, inspired by: -Basic training in palliative care for volunteers, offered by the Palliative Networks in Flanders [41] - Course book on dealing with existential questions [42] - ‘Volunteers as referrers’ (Christian Sickness Fund) [43]

Participants will have increased awareness of the support network and support needs of the resident.	- Gaining insight into the social network around the community resident and the place of a volunteer in that network - Gaining insight into where improvements or action points regarding communication lie within the network around the resident	Social ecological mapping exercise **(**Appendix IV**)**

Participants will have increased skills and self-efficacy in communicating and addressing identified support needs with informal caregivers and healthcare professionals.	- Introducing the topic and expressing attitudes and opinions - Encouraging assertive self-introduction by the volunteer to present themselves to healthcare professionals and relatives - Increasing awareness of making agreements about communication	Social ecological mapping exercise **(**Appendix IV**)**, Volunteer introduction flyer **(**Appendix V**)** and Role play exercises, inspired by basic training in palliative care for volunteers, offered by the Palliative Networks in Flanders [41]

Participants will have increased skills and self-efficacy in communicating and addressing identified support needs with community residents.	- Increasing awareness of the importance of consulting the community resident - Increasing awareness of the importance of confidentiality and ethics - Knowing and being able to apply the 4O model (translation from Dutch) to explore care signals	Role play exercises (Appendix IV), inspired by: - Basic training in palliative care for volunteers, offered by the Palliative Networks in Flanders [41] - Course book on dealing with existential questions [42] - ‘Volunteers as referrers’ (Christian Sickness Fund) [43] - SEE ME training [44] - An Katz's communication model (4O model) [45]

**PART 2: follow-up session (3 h)**	All	All	Discussion tables

#### 3.1.4 Volunteer consultation

We sought feedback on the design, content and materials of the workshop from community-based volunteers. While we contacted 14 volunteers who expressed interest, ultimately only one volunteer participated in the consultation. This feedback was helpful in revising the materials in terms of simplified instructions and avoiding excessive academic language. Additionally, the volunteer suggested to provide time for consolidation and reflection on the insights gained following each exercise in the workshop. Finally, regular breaks was also integrated into the workshop scheduling following input from the volunteer.

### 3.2 Program production (Step 4 of IMP)

#### 3.2.1 Pretest and refining of workshop materials

The workshop components and materials were evaluated for face and cognitive validity with six colleagues from the End-of-Life Care Research Group (UGent) and with eight stakeholder advisory board members. They were evaluated in terms of their clarity and difficulty of instructions, working methods, and content. This lead to further streamlining of the workshop language and content. The duration of the didactic workshop was extended from six to 7.5 h to accommodate a welcome, introduction, scheduled breaks, and a closing session. The quiz on general misconceptions about palliative care was complemented with additional clarification following the presentation of each question. It was also decied that participants were permitted to respond to the questions as a team to prevent any potential feelings of intimidation or vulnerability. While testing the exercises, several community resources were identified that could be shared with volunteers upon completion of the exercises. Additionally, a new section was added to the training materials, focusing on the boundaries of the volunteer role and the importance of volunteers being able to identify and access appropriate support.

#### 3.2.2 Production of workshop program

The Attentive Visitors workshop was developed as a two-part workshop, comprising a didactic workshop (7,5 h) and a follow-up session (3 h) organized approximately two months later. The didactic workshop provides knowledge about palliative care and palliative care needs, insights into the role and support of the social network, and communication skills to facilitate discussions and explore care needs with the community residents and healthcare professionals. The follow-up session focuses on group reflection (i.e., how have the volunteers integrated what they've learned into practice, what new or persistent challenges have they faced) and knowledge exchange (i.e., enabling volunteers to gain insight and advice from one another on the challenges they encounter in their volunteer roles). The follow-up session addresses the need for reflection, intervision and sharing of experiences and is designed to be repeatable over time (e.g., every few weeks or months). It provides an opportunity to consolidate the newly acquired knowledge and skills from the didactic workshop by briefly revisiting them, thereby increasing their chance of being implemented. Finally, the optimal group size was established through consultation with our stakeholder advisory board, and the workshop was designed for groups of 10–15 participants.

### 3.3 Overview of workshop program

Table 3 provides a comprehensive overview of the various components, including learning objectives, sub-goals and corresponding methods, that constitute the final two-part ‘Attentive Visitors’ workshop for community volunteers.

Appendix VIII provides a comprehensive and detailed elaboration of the contents of Table 3. Additionally, Appendix VIII outlines how learning objectives, sub-objectives, and methods are implemented in the Attentive Visitors workshop.

## 4 Discussion

This study describes the development and content of the Attentive Visitors workshop, an evidence-informed training designed to support community volunteers in their palliative care signposting role. By integrating a 7.5 h didactic workshop with a 3-h follow-up session, the workshop aims to (1) increase awareness of the volunteer's role and improve understanding of the support network and support needs of the community residents; (2) Expand knowledge about palliative care needs and available resources; and (3) Strengthen communication skills and self-efficacy for engaging with community residents, healthcare professionals, and identifying available community and professional care resources. The workshop incorporates experiential learning methods to encourage practical application in real-life volunteer activities.

## 5 Strengths and limitations

First, to our knowledge, this study is the first to develop a workshop tailored to equip community volunteers with knowledge and skills to recognize, describe and signpost palliative care needs to healthcare professionals. The evidence-informed Attentive Visitors workshop adds value to current experiences of communication and information sharing between community volunteers and healthcare professionals. Second, the use of the Intervention Mapping protocol, ensured a structured, theory- and evidence-based approach, integrating stakeholder and end-user perspectives. Third, the diversity in backgrounds, perspectives, and expertise, and the commitment of our stakeholder advisory board to the co-development of the workshop ensured that workshop components were relevant in addressing community volunteers needs and gaps in existing training programs. This co-development process enhances the workshop's feasibility and potential effectiveness for real-world implementation. Another strength is the workshop's alignment with the Compassionate Communities framework. By fostering skills that encourage connection, communication, and collaboration, the workshop empowers volunteers to strengthen social networks and improve information exchange with healthcare professionals. These contributions are critical in bridging gaps in support for individuals with palliative care needs. Despite its strengths, the study has limitations. Input from community volunteers was limited due to challenges in bringing together participants, including holiday plans, volunteer responsibilities, and caregiving commitments, resulting in feedback from only one volunteer. Although initially interested, many volunteers disengaged when repeatedly asked for their availability digitally. One-on-one consultations or alternative engagement strategies might have been more effective in capturing their perspectives. An additional consideration concerns the time investment required for the full-day workshop. The limited response from volunteers during development may indicate a participation barrier, given their other commitments such as work or other caregiving responsibilities. This raises concerns about the workshop's feasibility and scalability. It is recommended that this aspect be explicitly investigated during pilot testing and evaluation, with particular focus on accessibility and conditions for implementation. Finally, only Flemish training programs for community volunteers on signposting and communication were reviewed. The search for existing training courses was neither systematic nor exhaustive. Reviewing, adapting, and translating international training courses was beyond the scope of this study; however, future research could benefit from comparing the Attentive Visitors workshop with international training programs.

### 5.1 Innovative aspects of the “Attentive visitors” workshop

#### 5.1.1 Fit within the Compassionate Communities movement

The workshop's alignment with the Compassionate Communities movement [48,49] is a key innovation. By equipping community volunteers to recognize, describe, and address palliative care needs, it extends their capacity to support community residents, fosters stronger connections between informal and professional care networks, and enhances communication and collaboration with other stakeholders involved in the care process. The Attentive Visitors workshop emphasizes capacity building by enhancing the skills and confidence of volunteers, fostering stronger connections within the community to ensure more effective and sustainable support. Furthermore, the workshop values the experiences of volunteers and utilizes them as a valuable 'asset' to support other volunteers, including through the organization of a follow-up session.

Another key component of Compassionate Communities approaches is to develop a way to connect and enhance the social support and care networks that already exist in community life [48,49]. The Attentive Visitors workshop contributes to this by aiming to connect and enhance the interactions between volunteers and generalist primary care services by supporting volunteers' abilities to recognize, describe, and communicate care needs to the person with care needs and professional caregivers. The Attentive Visitors workshop also aligns with the core ideas behind Abel et al.’s ‘Circles of Care’ model [50] which applies a socio-ecological model to end-of-life challenges, viewing individuals, communities, service organizations and policy makers working together to provide end-of-life care that increases the value and meaning for people at the end of their lives, both patients and communities [50].

#### 5.1.2 Focus on signposting

While other programs have focused on communication skills [29,[31], [32], [33], [34]], grief [33], spiritual diversity [31], symptoms [32], self-care [29,31,32] and care navigation [13,14], the Attentive Visitors workshop uniquely emphasizes the signposting role of volunteers, which is an area often overlooked. The workshop focusses on strengthening self-confidence and self-efficacy, which is a critical gap in volunteer training. Additionally, the attentive Visitors workshop is co-developed and evidence-informed, building on a qualitative needs assessment study that includes input and perspectives from multiple stakeholders. The accessible nature, concise format (short duration of 1.5 days) and modular structure make it complementary to existing training programs, ensuring accessibility and relevance for diverse audiences.

#### 5.1.3 Experiential learning

The Attentive Visitors workshop integrates David A Kolb's experiential learning cycle, encompassing experiencing, reflecting, thinking, acting [51]. The methods used in the Attentive Visitors Workshop are experiential learning activities. These hands-on, interactive and collaboratice exercises create opportunities to connect theory to real-life case studies, encouraging community volunteers to actively engage, reflect, and apply their learning in their volunteer work. The organization of a follow-up moment serves as a practical application of the experiential learning cycle. Community volunteers are encouraged to share and reflect on experiences and challenges they've encountered in their volunteer work since the first workshop day. By attending the follow-up session, community volunteers are encouraged to integrate and apply their newly gained insights into their ongoing volunteer activities, completing the cycle of learning through experience, reflection, thinking and acting. The focus on peer learning and experiential learning within the Attentive Visitors workshop aligns with the idea of a community of practice in which community volunteers can share knowledge, expertise and experiences, learning from each other to address challenges they encounter [52]. The developers of the Attentive Visitors workshop encourage the continued organization of follow-up moments after the Attentive Visitor Workshop has been completed.

### 5.2 Recommendations for policy, practice and further research

In the future, the Attentive Visitors training program could become part of the standard training curriculum for community volunteers, offered through health insurance funds or other training providers. However, before implementation, future research should pilot-test and evaluate the Attentive Visitors Workshop regarding effectiveness, acceptability, and feasibility before wider implementation. This includes assessing its alignment with the needs of community volunteers, community residents, and informal caregivers, and determining whether program adjustments are needed. Evaluating its impact on improving volunteers’ death literacy would also provide valuable insights. Developing such a training requires collaboration with stakeholders, including end-users and future implementers, ensuring programs are co-created through open dialogue and mutual respect. Managing power dynamics, along with fostering collaboration and reciprocity, is essential for successful co-creation. Policymakers must recognize community volunteers as key partners in palliative care and prioritize their training and support. With the growing global aging population, equipping volunteers for palliative homecare is an urgent international priority.

## 6 Conclusion

In line with public health approaches to connect and enhance the social support and care networks that already exist within communities – exemplified in the Compassionate Community movement, an evidence-informed workshop was developed supporting community volunteers in their signposting role, with a focus on enhancing connection, collaboration, communication and information exchange between volunteers and healthcare professionals. In the future, the Attentive Visitor workshop will be pilot tested and evaluated to assess its effectiveness, acceptability, and feasibility.

## Author statements

- Sabet Van Steenbergen: Conceptualization, Data curation, Formal analysis, Investigation, Writing - original draft, Writing – review and drafting.

- Steven Vanderstichelen: Conceptualization, Data curation, Funding acquisition, Investigation, Methodology, Project administration, Supervision, Writing original draft, Writing - review & editing.

- Else Gien Statema: Review & editing.

- Luc Deliens: Supervision, Writing - review & editing.

- Sarah Dury: Supervision, Writing - review & editing.

- Kenneth Chambaere: Conceptualization, Data curation, Funding acquisition, Investigation, Methodology, Project administration, Supervision, Writing - review & editing.

## Ethical approval

The research project that his study was part of received ethical approval from the Medical Ethics Committee of the Ghent university Hospital (Ghent University) on November 10, 2020 (B U N.: B6702020000359). An amendment prolonging the study period due to COVID-19 June 30, 2022 was approved on December 15, 2021.

## Availability of data and materials

The data and materials of this study are with the first author and are available upon reasonable request.

## Funding

This work was supported by the Flemish Cancer Fund (Kom op tegen kanker) under grant nr PSC 17/6/19–000140946 and The Flemish Research Foundation (FWO) under grant nr. 1200424N.

## Declaration of competing interest

The authors declare that they have no known competing financial interests or personal relationships that could have appeared to influence the work reported in this paper.
