# Supplementary material for: Development of the ‘Attentive Visitors’ workshop supporting community volunteers in their palliative care signposting role
Source: Public Health Pract (Oxf). 2025 Oct 4;10:100663. doi: 10.1016/j.puhip.2025.100663 (PMC12547292; doi:10.1016/j.puhip.2025.100663)
Supplement: Multimedia component 1 [file mmc1.docx]

**Appendix I: examples of pictures used in the introduction exercise of the didactic workshop**


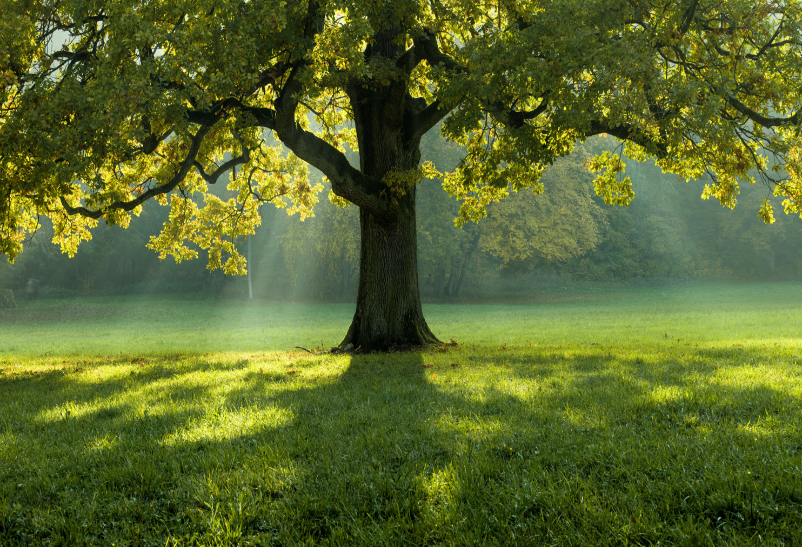

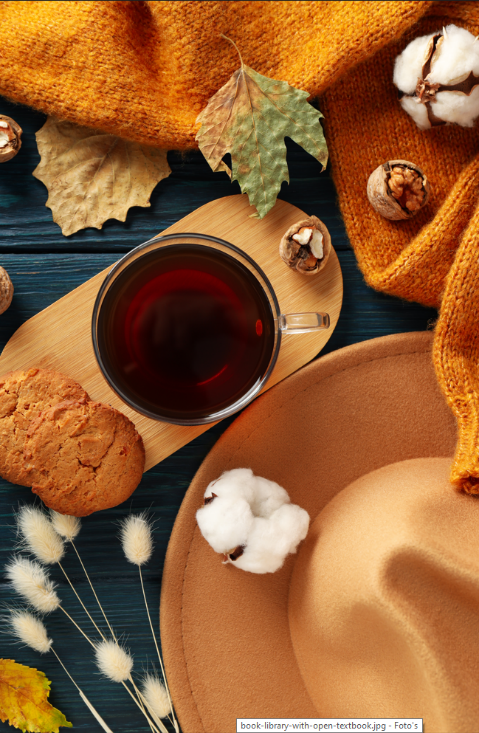


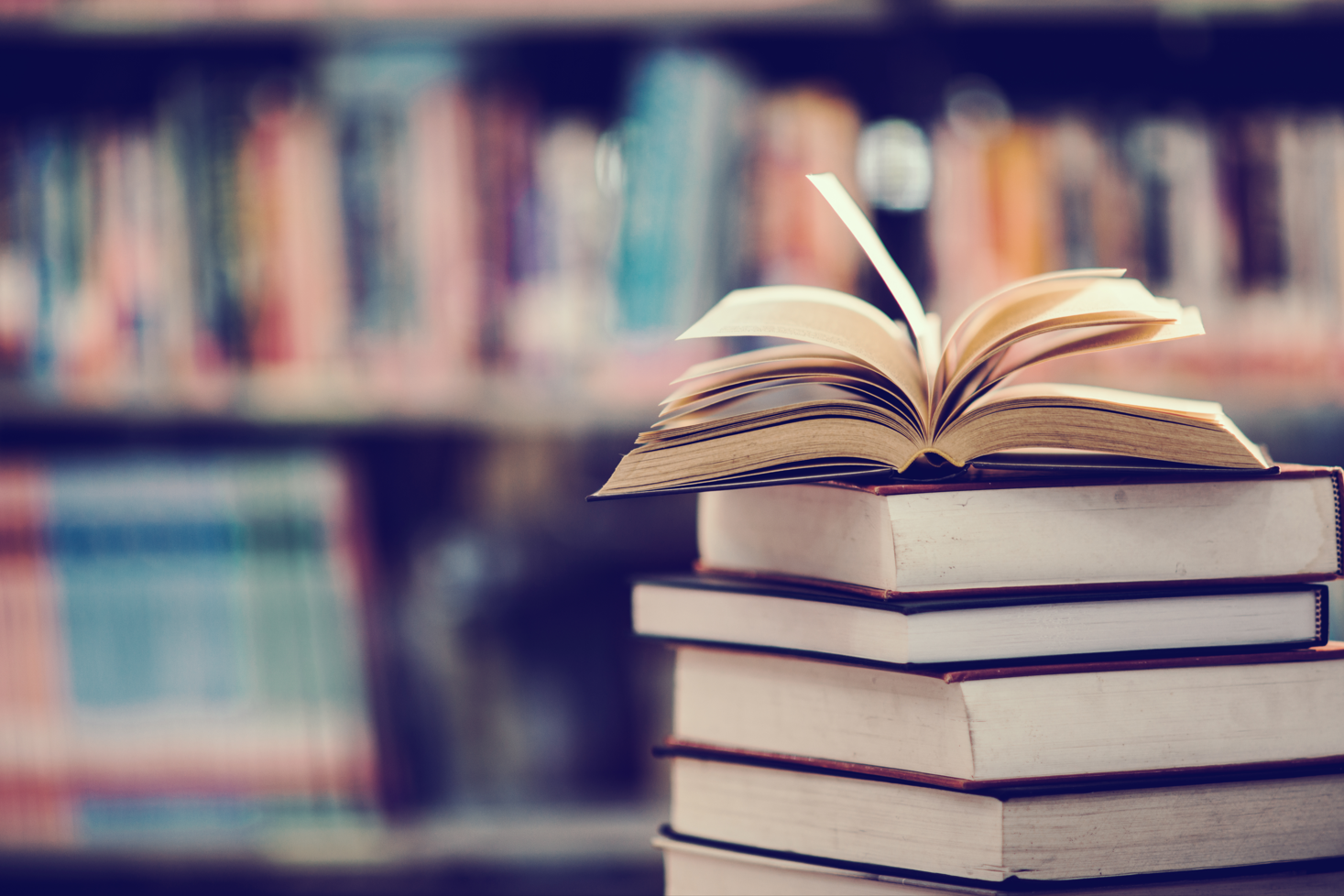


**Appendix II: quiz**

**Question 1: We can conclude that the terms ‘palliative care’ and ‘terminal care’ are often used interchangeably. Which of the following statements is correct?**

A. Terminal care is a synonym for palliative care.

B. Palliative care starts with a life-threatening diagnosis and ends with terminal care.

C. Palliative care only starts when treatment is stopped.

**Question 2: Palliative care can provide an answer to the following care needs:**

A. Physical and practical needs (pain, fatigue, reduced mobility, etc.)

B. Psychosocial and existential needs (dealing with fears, dealing with grief, dealing with questions about meaning, etc.)

C. A total package of care needs consists of physical and practical care needs, social care needs, psychological care needs and existential care needs.

**Question 3: from what point is palliative care best deployed?**

A. Palliative care is used in a timely manner from the moment someone is diagnosed with a life-threatening illness.

B. Palliative care is used when one would not be surprised if the neighbour would die within a year (= surprise question)

C. Palliative care is used from the moment the neighbour asks for it.

**Question 4: Which answer about the target group of palliative care is the most correct?**

A. Palliative care focuses on patients with an incurable condition

B. Palliative care focuses on patients and informal caregivers

C. Palliative care focuses on the local resident with an incurable condition and the entire care network.

**Question 5: You come to a neighbor's home as an attentive visitor. The nurse is sitting at the table and feeding the neighbor. What can you do in this situation?**

A. I take over from the nurse and feed the neighbor.

B. I also sit at the table and start a conversation with the neighbor.

C. Afterwards, I start a conversation with the nurse and exchange contact details.

**Question 6: What is true about advance care planning?**

A. You only have one conversation about advance care planning, so you have to know exactly what you want.

B. Only a professional caregiver may have a conversation about advance care planning.

C. A conversation about advance care planning is about values, wishes and preferences with regard to future care and death.

**Appendix III: case studies**

| **CASE STUDY 1: LODE**  Male, Lode, 84 years old. Married to Maria, 85 years old, 3 children, they have almost no contact with the children.  Lode has always been an active man: hard work and several hobbies outside of that, including gardening and cycling. Lode is often alone because that is when “he can really relax”. He now also goes for short bike rides, and often walks around in the garden that is no longer maintained. He has never been a talker, certainly not about his own feelings, concerns.  Lode was diagnosed with Parkinson's disease 10 years ago. Initially he only had mild symptoms. For 1 year now he has noticed a faster physical decline. He can just about get up from a chair by himself, he needs help from the couch. He is also partly dependent for his daily activities, e.g. help with dressing and undressing (buttons, belt, ...). Recently there has also been incipient forgetfulness and loss of concentration.  Lode refuses all (professional) help: his wife will do everything. One of the children goes to do the big shopping every two weeks, Maria does the smaller shopping every two days.  Maria worries when she leaves him home alone: ​​will accidents happen? Will he get to the toilet in time when he needs to? And so on. Because of these worries, she goes out less often, and for example, she hardly goes to her weekly card club anymore.  Maria is tired, she has very little rest because of the continuous care for Lode. On the one hand, she longs for rest, on the other hand, she wants to take care of him and respect him in his refusal of professional home support.  A friend of hers told me about volunteers who come to keep someone company for a while and who can also offer a listening ear to the informal caregivers. This is how you come to visit for the first time. |
| --- |

| **CASE STUDY 2: MIEKE**  Woman, Mieke, 72 years old, widow for 4 years, no children.  Mieke has been a socially active woman. She always went to activities of the parish and of Femma: the cooking club and the sewing salon, the first because she loves to cook, the second more for the fun. After her husband died four years ago, many friends stopped visiting.  Mieke has had colon cancer for 3 years, and has had an operation and chemotherapy for it. Since then she has been living with a stoma.  Lately she has become weaker, she has been told by the specialist that there are metastases in the liver and lungs. The cancer can no longer be cured, but can still be slowed down somewhat with palliative chemotherapy. If nothing is done, according to the doctor she will live a maximum of six months.  Mieke doubts whether she will start the palliative chemo. The doctors cannot say how long she will have. She is afraid of the side effects and further weakening due to the therapy.  In her daily life, Mieke is ashamed of her stoma: she has the impression that it often smells. She knows rationally that this is not the case, but still… She is depressed. If she can no longer ‘go outside’, to activities, then she just sits at home alone and waits for the inevitable. Then she no longer needs it.  You and your colleagues have been coming by as volunteers for a while now to keep Mieke company and to help. |
| --- |

**Appendix IV: Stakeholder map**


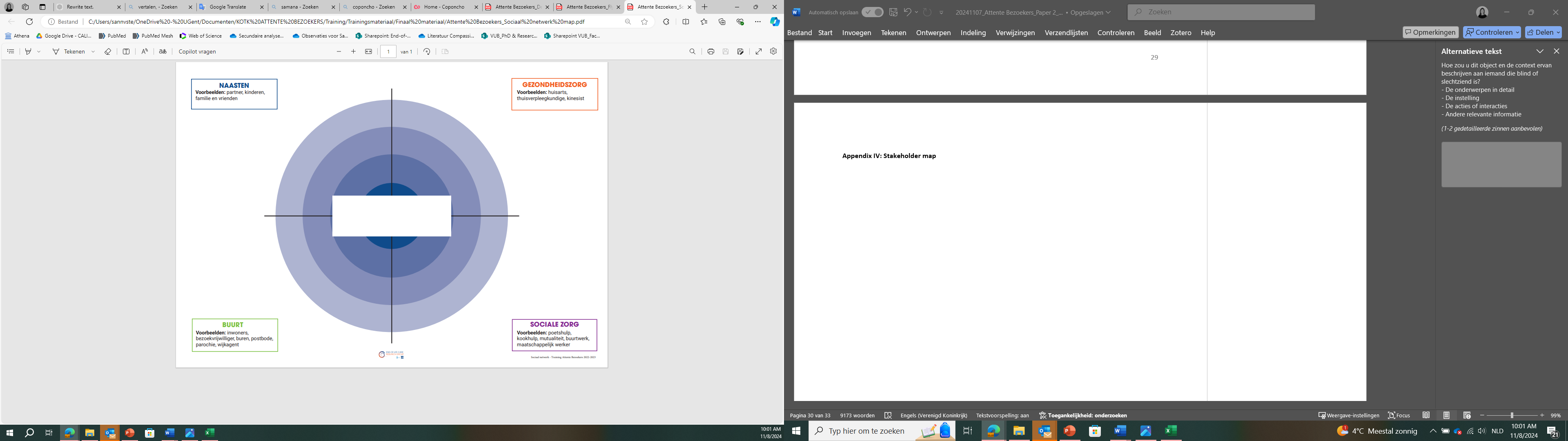


**Appendix V: flyer**


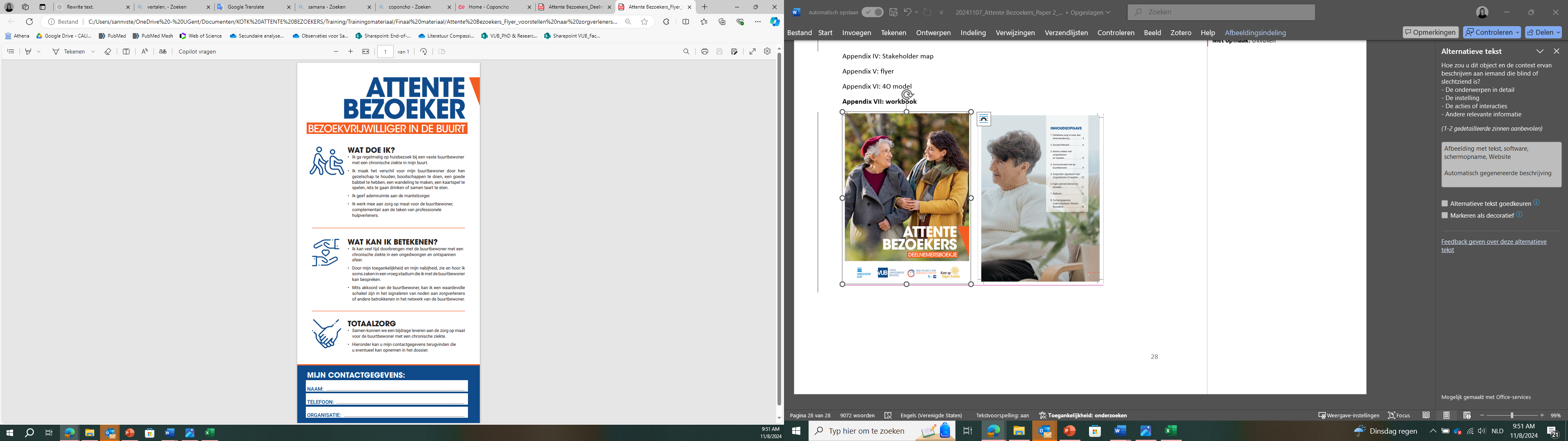


**Appendix VI: 4O model**

| **O – Opening statement** | Name very literally what you have seen or heard:   - 'I see that there are still many thresholds here at home' - 'I understood that you like to go to Femma activities, but you haven't been there in a long time?' |
| --- | --- |
| **O – Open Question** | What do you notice about this yourself? |
| **O – Options review** | Would it be useful if we asked if anything could be changed?' |
| **O – Follow up on concrete appointments** | Would you like to ask about this yourself? What help can I offer you?’  Then don’t forget to ask about it again during your next visit |

**Appendix VII: workbook**


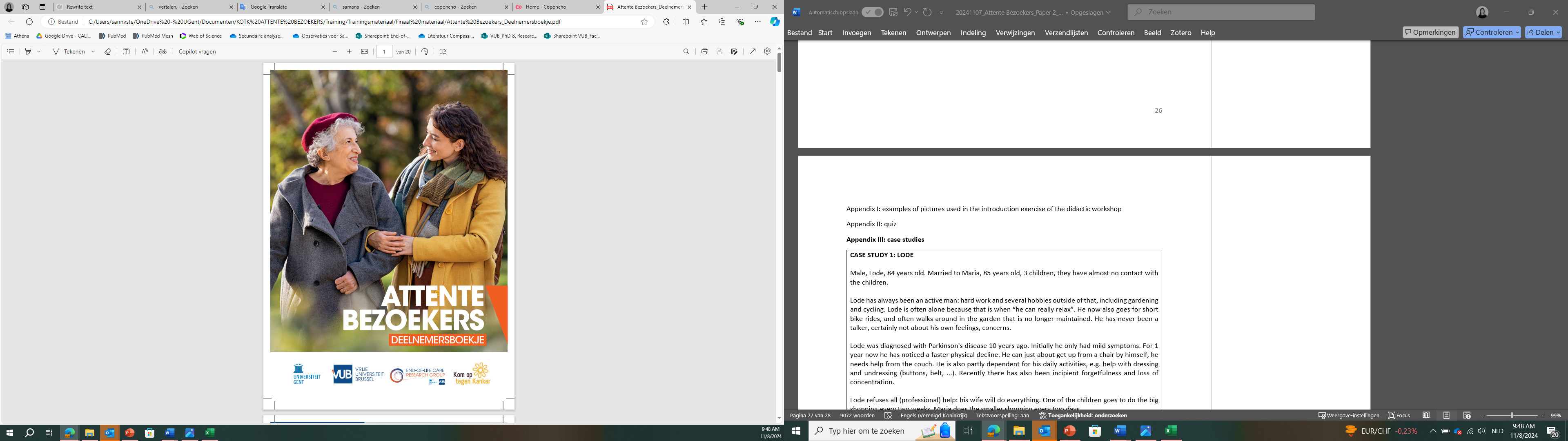

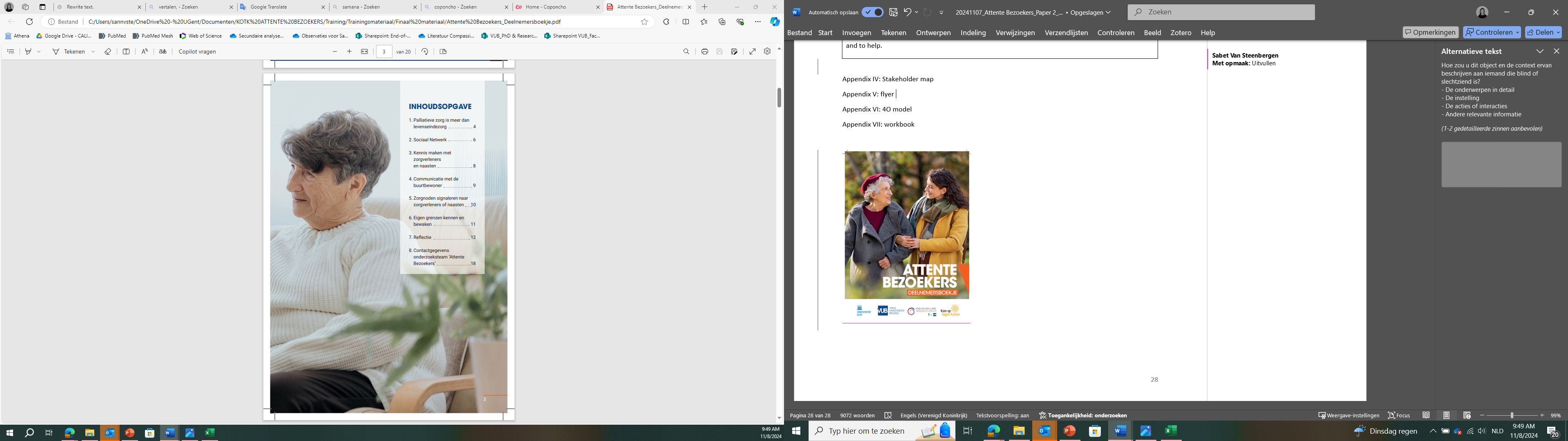


| **Appendix VIII: Learning objectives, sub-goals and corresponding methods used in the Attentive Visitors Worskhop** | | | | | |
| --- | --- | --- | --- | --- | --- |
|  | **Learning objective** | **Sub-goal** | **Method** | **Application in the Attentive Visitors workshop** | **Inspiration from review of existing training materials** |
| **PART 1: didactic workshop (7,5 hours)** | Participants will know their fellow participants better. | - Icebreaker - Getting to know eachother | Introduction by photos **(Appendix I)** | Participants are invited to select two that symbolize their role as a volunteer and to share with the group the reasons behind their choice. This activity allows the trainer to gain initial insight into how the volunteers perceive their roles, and it also serves as an opportunity for participants to become acquainted with one another. | Principles Photovoice^46^ and Photo Elicitation^47^ |
|  | Participants will have increased knowledge about palliative care. | - Activating and improving knowledge about palliative care - Reviewing and enhancing basic knowledge about palliative care - Increasing participants' understanding and knowledge of the various domains of palliative care and care needs | Quiz  **(Appendix II)** | The quiz questions explore participants' knowledge and perceptions regarding palliative care. Each question is thoroughly discussed, covering topics such as the distinction between palliative and terminal care, the types of care needs addressed by palliative care, the appropriate timing for involving palliative care, the target demographic for palliative care, and the concept of advanced care planning. | Basic training in palliative care for volunteers, offered by the Palliative Networks in Flanders^41^ |
|  | Participants will have increased awareness of the volunteer role and the signposting function. | - Increasing awareness of one's own volunteer role in palliative care - Increasing awareness of the potential signposting function of volunteers - Gaining insights into how to maintain one's own boundaries - Becoming aware of the importance of maintaining boundaries | Group discussion | Attention is paid to the unique and complementary contribution of volunteers to professional palliative care and their signposting role towards professional caregivers and family caregivers in recognizing and communicating care needs. Through a group discussion, participants explore who primarily provides complementary care and who identifies with the signposting role within the participant group. | - Basic training in palliative care for volunteers, offered by the Palliative Networks in Flanders^41^ - Dealing with existential questions, course book of Stephanie Vermeulen^42^ - Volunteers as referrers, offered by Christian Sickness Fund (CM)^43^ |
|  | Participants will have increased knowledge palliative care needs, and increased skills and self-efficacy in identifying available community and professional care resources. | - Increasing patricipants’ understanding and knowledge of the various domains of palliative care and care needs - Making connections between different forms of assistance and the various domains of palliative care - Gaining insight into one's tendencies as a volunteer and blind spots - Broadening our perspective on the dreams, wishes, hopes of community residents - Increasing knowledge about additional initiatives in the environment to support the community resident | - Case study **(Appendix III)** - Group discussion | The participants are prompted to identify potential actions they could take or questions they could ask in the presented situation. Subsequently, participants are required to identify the type of care need(s) that would be addressed by each action or question. This is achieved through the use of colored stickers, with each color corresponding to one of the four domains of palliative care. In the initial case study, participants engage in a reflective process, contemplating whether specific colors are more prevalent, absent, or if their responses demonstrate a tendency to prioritize certain care needs over others. Additionally, they assess their own attentiveness and the extent to which they may be more attentive to certain needs. The group then engages in a discussion of the case and the responses provided by the participants. In the second case study, participants commence in the same manner, contemplating actions or queries pertaining to the needs they have identified in the case study. Subsequently, they are furnished with supplementary data concerning the aspirations of the individual in question. Subsequently, participants are requested to re-evaluate their actions and questions in light of the newly provided information, which varies according to the specific group under consideration. The contrasts in methodology and outcomes between the two cases are then subjected to discussion, after which participants engage in reflection on the insights gained from these exercises. | SEE ME training^44^ |
|  | Participants will have increased awareness of boundaries and engaging other community resources. | - Becoming aware of the importance of maintaining boundaries - Gaining insights into how to maintain one's own boundaries - Increasing knowledge about how to identify additional initiatives in the neighborhood to support the community resident beyond the volunteer’s capacity | Group discussion | Based on the second case study, participants are divided into groups to explore the boundaries of what a volunteer can do, what they can’t do, and what they are willing to do. Recommendations are exchanged and provided for setting and maintaining boundaries without leaving people to their own devices. The focus then shifts to seeking additional help when meaningful and necessary (e.g., when the support needs of the community resident are outside of the scope of the volunteers’ support role) and exploring how to approach this. | - Basic training in palliative care for volunteers, offered by the Palliative Networks in Flanders^41^ - Dealing with existential questions, course book of Stephanie Vermeulen^42^ - Volunteers as referrers, offered by Christian Sickness Fund (CM)^43^ |
|  | Participants will have increased awareness of the support network and support needs of the resident. | - Gaining insight into the social network around the community resident and the place of a volunteer in that network - Gaining insight into where improvements or action points regarding communication lie within the network around the resident | Social ecological mapping exercise **(Appendix IV)** | In this section, participants learn to fill out a social map for a community resident. Various people in the immediate (social) environment of the community resident are placed on this map according to proximity and role, and attention is paid to the position and role of the volunteer on this map. The volunteer is asked to evaluate the network and estimate if more / strengthened (social) support or care would be helpful. They consider whom they have already met within the network, with whom they are actively communicating, and with whom it would be interesting to establish contact and why. |  |
|  | Participants will have increased skills and self-efficacy in communicating and addressing identified support needs with informal caregivers and healthcare professionals. | - Introducing the topic and expressing attitudes and opinions - Encouraging assertive self-introduction by the volunteer to present themselves to healthcare professionals and relatives - Increasing awareness of making agreements about communication | - Social ecological mapping exercise **(Appendix IV)** - Volunteer introduction flyer **(Appendix V)** - Role play exercises | Volunteers practice introducing themselves in the worst and beste possible ways to other professionals, caregivers, and relatives. Through a roleplay game with a voting juriy, volunteers are asked to introduce themselves succinctly yet clearly, enabling others to remember them and understand their significance to the community resident. At the end of the exercises, volunteers receive a flyer that can help them introduce themselves and that they can use freely in their volunteer work. | Basic training in palliative care for volunteers, offered by the Palliative Networks in Flanders^41^ |
|  | Participants will have increased skills and self-efficacy in communicating and addressing identified support needs with community residents. | - Increasing awareness of the importance of consulting the community resident - Increasing awareness of the importance of confidentiality and ethics - Knowing and being able to apply the 4O model (translation from Dutch) to explore care signals | Role play exercises **(Appendix IV)** | In this section, the 4O model is explained and applied in groups to the previous two case studies. The 4O model can be used to address difficult subjects or to explore care signals with the community resident. The model stands for: Opening statement, Open question, Options and Follow up (NL: Opvolging). | - Basic training in palliative care for volunteers, offered by the Palliative Networks in Flanders^41^ - Dealing with existential questions, course book of Stephanie Vermeulen^42^ - Volunteers as referrers, offered by Christian Sickness Fund (CM)^43^ - SEE ME training^44^ - An Katz’s communication model (4O model)^45^ |
| **PART 2: follow-up session (3 hours)** | All | All | Discussion tables | Volunteers are invited to share experiences and challenges they have faced in their volunteer work since participating in the first workshop. Participants collectively reflect on the relevance and practical application of the learned knowledge and skills since completing the didactic workshop module. The group rotates through four discussion tables in smaller groups. Each table focuses on a central aspect of the didactic workshop module: 1) needs and requirements of the community resident, 2) the social map and access to facilities, 3) communication with caregivers and relatives, and 4) communication with community residents. At each discussion table, participants are invited to choose a question or experience where they wanted to receive feedback from other volunteers in the group. Participants are encouraged to listen first, ask questions to explore the problem or challenge, and only then formulate advice. Finally, the person who posed the question summarizes everything before moving on to the next problem statement in the group. | Not applicable |
